# Morphometric Analysis of the Infraorbital Foramen: The Clinical Relevance

**DOI:** 10.1155/2016/7917343

**Published:** 2016-12-25

**Authors:** Deepthi Nanayakkara, Roshan Peiris, Navini Mannapperuma, Amal Vadysinghe

**Affiliations:** ^1^Division of Anatomy, Department of Basic Sciences, Faculty of Dental Sciences, University of Peradeniya, 20400 Peradeniya, Sri Lanka; ^2^Department of Forensic Medicine, Faculty of Medicine, University of Peradeniya, 20400 Peradeniya, Sri Lanka

## Abstract

The present study was conducted to ascertain the shape, size, presence of accessory foramina, direction, and the precise position of the infraorbital foramen (IOF) in relation to the inferior orbital margin (IOM), anterior nasal spine (ANS), nasion (Na), maxillary teeth, and supraorbital foramen/notch (SOF/N) in adult skulls in a Sri Lankan population. Fifty-four skulls (42 males and 12 females) were analyzed. The IOF was oval in shape (38.6% and 36.3% on the right and left side, resp.) in a majority of skulls. The direction of the IOF was mostly medially downward (48.6%). Accessory foramina were found in 7.4% of the skulls. The infraorbital foramina were located at a mean distance of 6.52 ± 2.03 mm and 7.30 ± 1.57 mm, vertically below the IOM on the right and left side, respectively; 33.81 ± 2.68 mm and 34.23 ± 2.56 mm from the ANS on the right and left side, respectively; and 42.37 ± 3.52 mm and 42.52 ± 3.28 mm from the Na on the right and left side, respectively. In relation to the upper teeth the majority of IOF (37.5% and 55.9% on the right and left side, resp.) were located in the same vertical axis as the tip of the buccal cusp of the maxillary second premolar tooth.

## 1. Introduction

The infraorbital foramen (IOF) is located on the maxillary bone about 1 cm inferior to the infraorbital margin [1]. The infraorbital nerve and vessels are transmitted through this foramen. The infraorbital nerve, the continuation of the maxillary or second division of the trigeminal nerve, is solely a sensory nerve. It traverses the inferior orbital fissure into the inferior orbital canal and emerges onto the face at the IOF. It divides into several branches that innervate the skin and the mucous membrane of the midface, such as the lower eyelid, cheek, lateral aspect of the nose, upper lip, and the labial gum [1].

The IOF is an important landmark in facilitating anaesthetic and surgical interventions of the midface region. The infraorbital nerve block is widely used to accomplish regional anaesthesia during surgeries involving the midface region and paranasal sinuses [2, 3]. Traumatic or iatrogenic injury to the infraorbital neurovascular bundle may result in bleeding and hypoesthesia or paraesthesia or anaesthesia in the region of its supply [4]. Hence, detailed knowledge of the precise anatomical location and the possible variations of the IOF is fundamental to ensure safe and successful regional anaesthesia and to avoid the risk of damaging the neurovascular bundle during surgery in this region.

Multiple studies have demonstrated that the dimensions and relative position of the IOF vary between the genders and among different population groups [5–22]. To ascertain its precise location various soft tissue and bony landmarks have been utilized. Significant variations have been reported in the literature with regard to the position of IOF in relation to the infraorbital margin [3, 5, 6, 8, 14]. Moreover, the position of IOF in relation to maxillary teeth has been shown to vary among population groups [3, 13, 21].

Despite its clinical relevance, information available on the dimensions and relative position of the IOF in the Sri Lankan population is scarce. Hence, the present study was undertaken to ascertain the shape, presence of accessory foramina, direction, and the dimensions of the IOF and its position in relation to clinically relevant anatomical landmarks.

## 2. Material and Methods

Fifty-four adult dry skulls from 42 male and 12 female skeletons formed the study material. Skulls were collected from the Division of Anatomy, Department of Basic Sciences, Faculty of Dental Sciences, University of Peradeniya, Sri Lanka. Approval for this study has been granted by the Faculty Research Committee of the Faculty of Dental Sciences, University of Peradeniya (number FDS-FRC/2014/06). The skulls of known sex and age with no apparent gross pathology, deformity, or traumatic lesions were included in the study. The skulls with alveolar bone resorption and those of less than 18 years of age were excluded. The skulls with damage in the orbital and nasal cavity region were also excluded.

Both sides of the skulls were visually assessed and the shape, presence of accessory foramina, and the direction of the IOF were recorded. The shape of the IOF was described as displaying an oval, triangular, semilunar, or a circular outline. The direction of the opening of the infraorbital canal through the anterior surface of the maxilla was determined by inserting a flexible wire and recorded as being downward, medially, or medially downward.

**Figure 1:**
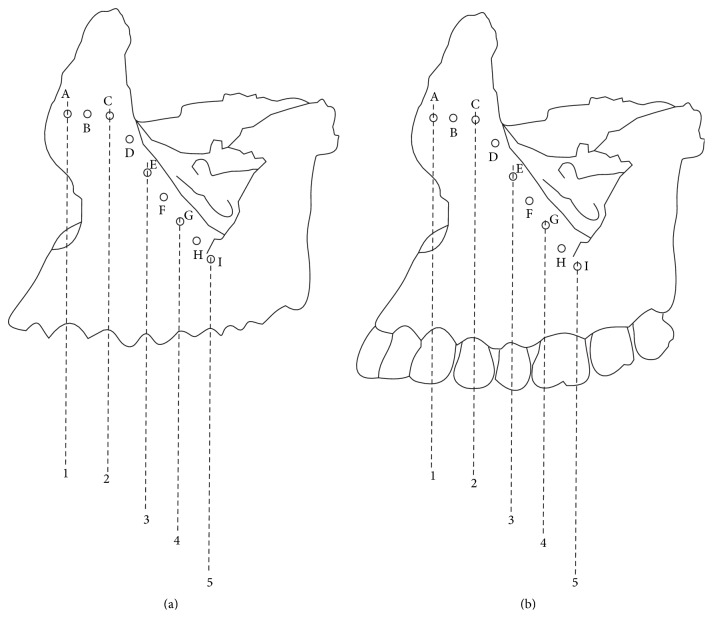
Relative position of the IOF in relation to maxillary teeth. (a) Relative position of IOF in an edentulous maxilla. (b) Relative position of IOF in a dentulous maxilla. 1: line passing through the cusp tip/middle of the buccal socket margin of the maxillary canine. 2: line passing through the buccal cusp tip/middle of the buccal socket margin of the maxillary first premolar. 3: line passing through the buccal cusp tip/middle of the buccal socket margin of the maxillary second premolar. 4: line passing through the cusp tip/middle of the buccal socket margin of the mesiobuccal cusp of the maxillary first molar. 5: line passing through the cusp tip/middle of the buccal socket margin of the distobuccal cusp of the maxillary first molar.

The relative position of the IOF in relation to the upper teeth was recorded either as in line with the same vertical axis passing through the cusp tip of the upper canine, or buccal cusp tip of the upper first premolar, or second premolar, or mesiobuccal and distobuccal cusp tips of the first molar or as lying in the vertical axis passing between the canine and the first premolar or first and second premolar or second premolar and first molar (Figure 1). In the presence of a tooth socket, the vertical axis was determined as the line passing through middle of the buccal socket margin of the tooth (Figure 1). The relative position of the IOF in relation to the supraorbital foramen or supraorbital notch (SOF/N) was recorded as lying in the same vertical plane as the SOF/N or lying lateral or medial to this plane.

**Figure 2:**
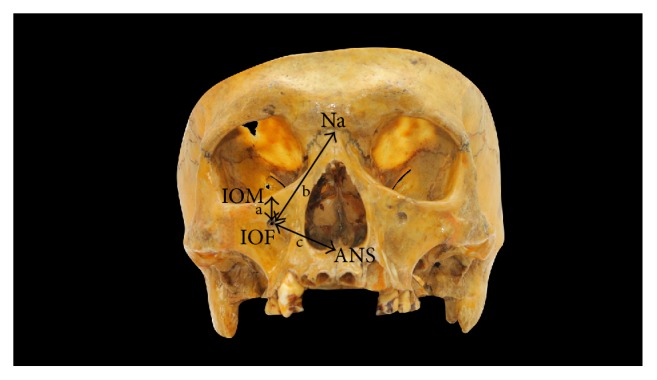
Measurements taken to determine the position of the IOF. IOM: inferior orbital margin, Na: nasion, and ANS: anterior nasal spine. a: the vertical distance between the IOF and IOM. b: the distance between the IOF and Na. c: the distance between the IOF and ANS.

In order to analyze the size and the relative position of the IOF, the following parameters on the right and left sides were measured using a digital vernier caliper to the nearest 0.01 mm (Mitutoyo, Japan):

1. The maximum vertical diameter of the IOF.
2. The maximum horizontal diameter of the IOF.
3. The vertical distance between the IOF and the inferior orbital margin (IOM) (Figure 2).
4. The distance between the IOF and the anterior nasal spine (ANS) (Figure 2).
5. The distance between the IOF and the nasion (Na) (Figure 2).

In order to minimize the intraobserver error, all measurements were recorded by one investigator. Three repeated measurements were made for each observation at different sittings and the average of the three measurements was taken for further analysis.

Results were expressed as means and SDs and the differences in the size and location of IOF between the left and right side and male and female were analyzed using the Statistical Package for Social Sciences (SPSS), 19th version. Students' *t*-test was used for the analysis and *P* < 0.05 was considered as statistically significant.

## 3. Results

An IOF was present on both sides of all skulls examined. A single IOF was seen in 92.6% and accessory foramina were found in 7.4% of the skulls. Meanwhile, one skull showed bilateral double foramina (1.8%). The predominant shape of the IOF was oval (38.6% in the right side and 36.3% in the left side), followed by semilunar (29.6% in the right side and, 27.6% in the left side), triangular (18.2% in the right side and 19.1% in the left side), and circular in outline (13.6% in the right side and 17.0% in the left side) (Table 1). The opening of the IOF was directed medially downward in 48.6%, medially in 43.3%, and downward in 8.1% of the skulls observed.

The vertical and transverse diameters of the IOF and the linear distances from the IOF to selected anatomical landmarks, IOM, ANS, and Na are shown in Tables 2 and 3. The size of the IOF and linear distances from the IOF to anatomical landmarks were larger in males than in females. However, the differences were not statistically significant except for the distance from the IOF to the IOM on the left side (*P* < 0.05) (Table 2). Further, the observed parameters were greater on the left side than those on the right. A statistically significant difference was observed only in the distance from the IOF to the IOM (*P* < 0.01) (Table 3) indicating that the IOF is located closer to the IOM on the right compared to the left. Furthermore, the left IOF had larger dimensions than the right IOF.

The relative position of the IOF in relation to the SOF/N is presented in Table 4. It is evident that the majority of infraorbital foramina are located lateral to the SOF/N (93.0% on the right side and 91.1% on the left side). Both IOF and SOF/N were located in the same vertical plane in 4.7% on the right side and in 8.9% on the left side of the total skulls.

The position of the IOF in relation to the maxillary teeth is shown in Table 5. It could be seen that the foramen was most commonly found in the vertical plane passing through the tip of the buccal cusp of the second premolar on both the right (37.5%) and left (55.9%) sides.

## 4. Discussion

The infraorbital nerve, which emerges through the IOF to appear on the face, is responsible for the sensory innervation to the skin of the malar area between the lower eyelid and the upper lip [1]. Since the infraorbital nerve provides a considerably large area of sensory innervation, it is a prime candidate for a regional nerve block. It is important to identify the infraorbital neurovascular bundle during surgery involving the midface and maxillary sinuses and when administering the infraorbital nerve block because injury to infraorbital neurovascular bundle carries a significant morbidity including numbness of the upper lip, lateral wall of the nose, lower lid, and the infraorbital region of the affected side and may pose significant implications to the patient's quality of life [4]. The present study indicates that the location and morphology of infraorbital foramen are asymmetrical and varied between males and females. The ability to reliably and accurately locate the IOF is therefore essential in the fields of dentistry, maxillofacial surgery, and otolaryngology alike and, consequently, has long been an area of interest of many scientists.

Numerous studies on the same topic have documented various soft tissue and bony landmarks [5–22] that would assist clinicians in identifying the location of the IOF for infraorbital nerve block, orthognathic surgery, and reconstructive surgery. The IOM is the widely used anatomical landmark to predict the location of the IOF. A wide variation has been documented in determining the location of the infraorbital nerve and its foramen with regard to various anatomical landmarks [3, 5–16]. For example, Aziz et al. have measured the distance of IOF-IOM on 47 cadaveric heads and found the distance to be 8.5 ± 2.2 mm whereas Boopathi et al. studied the location of the IOF on dry skulls and reported the mean distance of the IOF from the inferior orbital margin as 6.57 ± 1.28 mm. Further, in a study by Apinhasmit et al. on Thai adult skulls it was found that the IOF was located 9.23 ± 2.03 mm below the infraorbital margin. The same study reported that the mean horizontal distance from the zygomaticomaxillary suture at the level of the infraorbital rim to the IOF was 2.15 ± 1.67 mm. However, palpating the zygomaticomaxillary suture is nearly impossible on the face of a patient even though it is readily observed on the dry skull. In the present study, the inferior orbital margin, nasion, and the anterior nasal spine which are palpable clinically on a patient were used to locate the IOF.

The present study demonstrates differences in the distances of the IOF-IOM, IOF-ANS, and IOF-Na in relation to gender endorsing findings of previous studies which have reported greater dimensions in males compared to females [3, 5, 6, 13, 15]. Further, these differences have been reported to be statistically significant in some previous studies [5, 6, 13] while others have reported the differences to be not significant [3]. This dimorphism in relation to gender is not an unusual finding as similar differences have been reported in other parts of the craniofacial complex [25, 26].

Interestingly, the position of the IOF in relation to IOM, ANS, and Na displayed side related differences in our sample, suggesting that the location of the IOF is not always bilaterally symmetrical in any one individual (Table 2). The present study revealed that the distance of IOF-IOM on the left side was significantly greater in males (7.66 ± 1.42 mm) than in females (6.38 ± 1.71 mm) (*P* < 0.05). Further, we found that the mean distances of IOF-IOM, IOF-ANS, and IOF-Na were greater on the left side than on the right side. However, only the difference in the distance of the IOF-IOM was found to be statistically significant in the comparison of values between sexes. Similar observations have been reported in other investigations conducted on Nigerian [11], Brazilian [16], and Indian [27] populations. Therefore, the present investigation together with findings from previous similar studies [11, 16, 27] emphasizes the fact that the IOF is situated closer to the IOM on the right side than on the left side. These findings are important considerations for surgeons operating on this anatomy in reducing the risk of injury to ION and when administering infraorbital nerve block.

Furthermore, the position of the IOF in relation to the IOM has been reported to vary approximately between 6 mm and a little over 10 mm in previous studies that compared different population groups (Table 6). The mean distances of the IOF-IOM in the present study, 6.52 ± 2.03 mm and 7.30 ± 1.57 mm on the right and left sides, respectively, are comparatively similar to those reported by Boopathi et al. [14] and Gupta [22] in Indian populations but higher values have been reported in other studies [3, 5, 9, 13, 15].

With regard to the distances from the IOF to ANS or IOF to Na, although they were greater in males when compared with females and greater on the left side of the crania than on the right side, no significant side or gender difference could be observed. These finding are in agreement with those reported in previous studies [11, 12, 24]. However, contrary to these findings, Agthong et al. [6] and Cisneiros de Oliveira et al. [15] reported that the values observed for the distance from the IOF to ANS varied significantly between the sexes and sides of the crania.

It could be seen that the distances between the IOF and ANS and IOF and Na differ among different population groups (Tables 7 and 8). These variations could be attributed to different population characteristics. The mean value of IOF-Na in the present analysis, which was 42.37 ± 3.52 mm on the right side and 42.52 ± 3.28 mm on the left side, is found to be lower when compared with those of other population groups [10, 24] (Table 8). The Na is a very important landmark in surgical anatomy to identify different anatomical locations. However, few studies are available in the literature to confirm its use in the identification of the IOF. Therefore, the present study provides significant information regarding the relationship of the IOF to the Na in a Sri Lankan population which could be useful for the surgeons.

It is evident that the distances IOF-IOM, IOF-ANS, and IOF-Na are variable among different world population groups. Moreover, our findings highlight substantial variability in the morphology and location of the IOF even within the Sri Lankan population, between males and females, and even between right and left sides within the same individual, a fact that emphasizes the significance of meticulous preoperative evaluation of the IOF in patients who are candidates for maxillofacial surgery and regional block anaesthesia [8].

The occurrence of accessory infraorbital foramina (AIOF) is well documented in the literature [3, 5–9, 13, 14, 28–33]. A wide variation in the occurrence of AIOF among different populations has also been reported. As reported by Leo et al. [28], the earliest account of variations of the IOF was given by Gruber in 1875. Gruber reported that the number of accessory foramina may vary from 1 to 5. Furthermore, Kadanoff et al. [29] studied 1400 skulls and reported the occurrence of 131 double (9%), 7 triple (0.5%), and 4 greater than three (0.3%) accessory foramina. Berry [30] studied AIOF in skulls from four geographical locations and the incidence of AIOF was reported to be 6.4% and 8.7% in Burmese males and females; 12.5% and 7.9% in North American males and females; 18.2% and 12.5% in Mexican males and females; and 2.2% and 4.8% in English males and females. In the present analysis the incidence of accessory infraorbital foramina was 7.4%. This incidence is comparatively similar to those reported by Tezer et al. [31] and Kazkayasi et al. [8] in Turkish populations. However, higher incidences have been reported in an Indian population (16.25%) by Boopathi et al. [14] and in Mexican males (18.2%) by Berry [30].

In addition, the occurrence of AIOF has been shown to vary on different sides of the cranium [31, 32]. Bressan et al. [32] reported the occurrence of AIOF as 4.7% in an Italian population and further identified that it is more common on the left side (2.16%) than on the right side (1.22%).

The presence of accessory foramina is important for surgeons because there may be an accessory branch of the infraorbital nerve passing through the AIOF. Duplication of the infraorbital neurovasculature has been reported in the literature and both the IOF and AIOF were observed to have their own individual neurovascular bundle [33, 34]. This knowledge is imperative for the surgeon dissecting the midface region in order to avoid iatrogenic injury to the duplicated infraorbital nerve and also to gain sufficient local anaesthesia [33]

The current study showed that, in a majority of the skulls, the infraorbital foramina were located lateral to the vertical plane passing through the SOF/N. The prevalence was 93.0% on the right side and 91.1% on the left side, a result that agrees with the findings of previous studies on Thai and Korean populations [5, 9]. In the meantime, in the present study, 4.7% and 8.9% infraorbital foramina on the left and right side, respectively, were seen to lie in the same vertical plane as SOF/N. Moreover, the occurrence of IOF in the same vertical plane as SOF/N was recorded as high as 23.4%, 38.1%, and 80% in Thai, Korean, and Indian populations, respectively.

As to the location of the IOF in relation to the maxillary teeth, it is important to note that the IOF was most frequently located in a vertical plane passing through the tip of the buccal cusp of the maxillary second premolar tooth (37.5% and 55.9% on the right and left side, resp.) followed by a position in between the first and second upper premolar teeth (34.4% and 26.5% on the right and left side, resp.). This observation supports the findings of previous reports showing that the IOF is commonly related to the vertical plane passing through the tip of the buccal cusp of the maxillary second premolar tooth [5, 23]. However, according to Aziz et al. [3], the maxillary tooth most commonly found in the same vertical plane as the IOF was the first premolar. In a small percentage of skulls the IOF was located in line with the maxillary first molar tooth (3.1% on the right side), a fact that merits caution as it is likely to be associated with the possibility of causing a failed infraorbital nerve block.

The infraorbital nerve is the nerve of choice for regional nerve block when performing surgeries in the orbital, buccal, and nasal areas. The infraorbital nerve block can be performed by accessing the nerve through an intraoral route or an extraoral route [35]. For these approaches, accurate localizing of the IOF is of prime importance. Once the location of the IOF is determined, the needle can be advanced either through the skin directly toward the IOF or through the mouth at the level of the incisor at alveolar-buccal mucosal margin in the subsulcal plane. Therefore, the information regarding the position of the IOF and its variations in the present Sri Lankan population will facilitate the successful placement of the infraorbital nerve block required for different maxillofacial surgeries. In addition, accurate identification of the IOF position is important for both diagnostic and clinical procedures. Clinically, nerves emerging from this foramen could probably be injured during surgical procedures, like orthognathic surgeries involving the correction of maxillary arch discrepancies, management of Le Fort II fractures, and so forth resulting in paraesthesia or anaesthesia. The IOF is also an important reference point used in orbital surgeries [7] and is an important surgical location for external access to the maxillary sinus (Caldwell-Luc operation) [36]. Furthermore, an understanding of the anatomical location of the IOF is of increased importance with the rising popularity of endoscopic procedures with limited visibility.

In the meantime, it is important to mention that the relatively smaller number of female skulls in this study was a major drawback and may have limited the ability to obtain statistically significant differences in relation to gender. Hence, further studies with larger samples are desirable.

## 5. Conclusion

This study specifically reports the characteristics and location of infraorbital nerve exits in a Sri Lankan population. The infraorbital nerve is the nerve of choice for regional nerve block when performing surgeries in the midface region. The ability to accurately localize the IOF is crucial to avoid injury to the infraorbital nerve. The presence of prominent ethnic variation and sex and side related differences in relation to the position of the infraorbital foramen indicate that extra care should be taken during surgical manipulation and administering regional nerve block in the infraorbital region to avoid surgical complications. Furthermore, the presence of accessory foramina has a clinical implication, as injury to any branch of the ION that exits through these foramina may result in sensory deficit. Therefore, the results of the present study in a Sri Lankan population have clinical importance when performing surgical procedures in the infraorbital region in order to prevent unnecessary complications.

## Figures and Tables

**Table 1:** Shape of the infraorbital foramen on the right and left sides of the crania (%).

Shape	Right %	Left %
Triangular	18.2	19.1
Oval	38.6	36.3
Semilunar	29.6	27.6
Round	13.6	17.0

**Table 2:** Measurements of the infraorbital foramina on left and right sides in males and females.

Measurement	Male			Female			*P* value
	Minimum	Maximum	Mean ± SD	Minimum	Maximum	Mean ± SD	
*Right infraorbital foramen*							
Maximum vertical diameter	1.46	3.69	3.06 ± 0.72	2.34	3.78	3.17 ± 0.51	NS
Maximum transverse diameter	2.10	3.83	3.21 ± 0.68	2.67	4.17	3.32 ± 0.50	NS
Distance from IOF to IOM	4.13	15.47	6.83 ± 1.97	3.28	9.25	5.52 ± 1.96	NS
Distance from IOF to ANS	28.54	40.47	34.25 ± 2.24	25.35	36.77	32.41 ± 3.56	NS
Distance from IOF to Na	33.27	53.37	42.70 ± 3.63	37.67	46.92	41.20 ± 3.00	NS
*Left infraorbital foramen*							
Maximum vertical diameter	2.42	4.06	3.51 ± 0.45	2.47	4.16	3.22 ± 0.61	NS
Maximum transverse diameter	2.55	4.47	3.55 ± 0.53	2.55	4.06	3.36 ± 0.59	NS
Distance from IOF to IOM	5.12	11.53	7.66 ± 1.42	3.38	8.67	6.38 ± 1.71	*∗*
Distance from IOF to ANS	28.60	39.76	34.41 ± 2.00	23.99	38.42	33.34 ± 4.05	NS
Distance from IOF to Na	34.22	49.37	42.79 ± 3.36	37.72	47.85	41.53 ± 2.92	NS

^*∗*^*P* < 0.05; NS: not significant.

**Table 3:** Descriptive data of infraorbital foramina on the right and left sides of the crania.

Measurement	Right			Left			*P* value
	Minimum	Maximum	Mean ± SD	Minimum	Maximum	Mean ± SD	
Maximum vertical diameter	1.46	3.78	3.11 ± 0.61	2.42	4.16	3.31 ± 0.55	NS
Maximum transverse diameter	2.10	4.17	3.27 ± 0.58	2.55	4.47	3.33 ± 0.59	NS
Distance from IOF to IOM	3.28	15.47	6.52 ± 2.03	3.38	11.53	7.30 ± 1.57	*∗∗*
Distance from IOF to ANS	25.35	40.47	33.81 ± 2.68	23.99	39.75	34.23 ± 2.56	NS
Distance from IOF to Na	33.27	53.37	42.37 ± 3.52	34.22	49.37	42.52 ± 3.28	NS

^*∗∗*^*P* < 0.01; NS: not significant.

**Table 4:** The relative position of the IOF in relation to the SOF/N.

Position	Right %	Left %
Same vertical plane as SON/F	4.7	8.9
Medial to SON/F	2.3	0.0
Lateral to SON/F	93.0	91.1

**Table 5:** Relative position of the IOF with regard to the maxillary teeth (%).

Position	Right	Left
A + B	0	0
C	9.4	8.8
D	34.4	26.5
E	37.5	55.9
F	15.6	8.8
G + H + I	3.1	0.0

**Table 6:** A comparison of mean distances from the IOF-IOM in different populations reported in previous studies.

Study	Distance from IOF-IOM (mm)
Apinhasmit et al., Thailand [5]	9.53 ± 2.23 males, 8.71 ± 1.51 females
Cisneiros de Oliveira et al., Brazil [15]	8.0 males, 8.0 females
Ilayperuma et al., Sri Lanka [13]	10.56 ± 1.74 males, 9.02 ± 1.58 females
Ukoha et al., Nigeria [11]	7.38 ± 2.28
Aziz et al., USA [3]	8.5 ± 2.2 males, 7.8 ± 1.6 mm females
Boopathi et al., India [14]	6.57 ± 1.28
Gupta, India [22]	7.00
Chung et al., Korea [9]	8.6
Elsheikh et al., Egypt [23]	6.37 ± 1.4 right, 6.7 ± 1.6 left
Present study	6.52 ± 2.03 right, 7.30 ± 1.57 left

**Table 7:** A comparison of mean distances from the IOF to the ANS in different populations.

Study	Distance from IOF to ANS (mm)
Cisneiros de Oliveira et al., Brazil [15]	36.0 males, 34.0 females

Ukoha et al., Nigeria [11]	29.01 ± 3.59

Lopes et al., Brazil [12]	34.70 ± 5.63 right side, 35.66 ± 3.91 left side

Ekambaram et al., India [20]	36.30 ± 2.26 right side, 36.00 ± 2.36 left side in males 34.31 ± 2.20 right side, 33.01 ± 2.31 left side in females

Agthong et al., Thailand [6]	34.8 right side, 35.0 left side in males 32.8 right side, 33.1 left side in females

Singh et al., India [24]	36.73 ± 3.11 right side, 36.51 ± 3.23 left side

Present study	34.25 ± 2.24 right side, 34.41 ± 2.00 left side in males 32.41 ± 3.56 right side, 33.34 ± 4.05 left side in females

**Table 8:** A comparison of mean distances from the IOF to Na in different populations.

Study	Distance from IOF to Na (mm)
Przygocka et al., Poland [10]	45.22 ± 3.20 right side, 44.375 ± 2.76 left side

Ekambaram et al., India [20]	38.45 ± 3.28 right, 37.95 ± 3.33 left side in males 32.86 ± 2.66 right side, 30.16 ± 2.06 left side in females

Singh et al., India [24]	45.23 ± 4.68 right side and 44.68 ± 4.59 left side

Present study	42.37 ± 3.52 right side and 42.52 ± 3.28 left side

## References

[1] Standring S. (2008). *Gray's Anatomy: Anatomical Basis of Clinical Practice*.

[2] Zide B. M., Swift R. (1998). How to block and tackle the face. *Plastic and Reconstructive Surgery*.

[3] Aziz S. R., Marchena J. M., Puran A. (2000). Anatomic characteristics of the infraorbital foramen: A Cadaver Study. *Journal of Oral and Maxillofacial Surgery*.

[4] Chandra R. K., Kennedy D. W. (2004). Surgical implications of an unusual anomaly of the infraorbital nerve. *Ear, Nose and Throat Journal*.

[5] Apinhasmit W., Chompoopong S., Methathrathip D., Sansuk R., Phetphunphiphat W. (2006). Supraorbital notch/foramen, infraorbital foramen and mental foramen in Thais: anthropometric measurements and surgical relevance. *Journal of the Medical Association of Thailand*.

[6] Agthong S., Huanmanop T., Chentanez V. (2005). Anatomical variations of the supraorbital, infraorbital, and mental foramina related to gender and side. *Journal of Oral and Maxillofacial Surgery*.

[7] Karakas P., Bozkir M. G., Oguz Ö. (2002). Morphometric measurements from various reference points in the orbit of male Caucasians. *Surgical and Radiologic Anatomy*.

[8] Kazkayasi M., Ergin A., Ersoy M., Bengi O., Tekdemir I., Elhan A. (2001). Certain anatomical relations and the precise morphometry of the infraorbital foramen-canal and groove: an anatomical and cephalometric study. *Laryngoscope*.

[9] Chung M. S., Kim H. J., Kang H. S., Chung I. H. (1995). Locational relationship of the supraorbital notch or foramen and infraorbital and mental foramina in Koreans. *Acta Anatomica*.

[10] Przygocka A., Podgórski M., Jędrzejewski K., Topol M., Polguj M. (2012). The location of the infraorbital foramen in human skulls, to be used as new anthropometric landmarks as a useful method for maxillofacial surgery. *Folia Morphologica*.

[11] Ukoha U. U., Umeasalugo K. E., Udemezue O. O., Nzeako H. C., Ndukwe G. U., Nwankwo P. C. (2014). Anthropometric measurement of infraorbital foramen in south-east and south-south Nigeria. *National Journal of Medical Research*.

[12] Lopes P. T. C., Pereira G. A. M., Santos A. M. P. V., Freitas C. R., Abreu B. R. R., Malafaia A. C. (2009). Morphometric analysis of the infraorbital foramen related to gender and laterality in dry skulls of adult individuals in southern Brazil. *Brazilian Journal of Morphological Sciences*.

[13] Ilayperuma I., Nanayakkara G., Palahepitiya N. (2010). Morphometric analysis of the infraorbital foramen in adult Sri Lankan skulls. *International Journal of Morphology*.

[14] Boopathi S., Chakravarthy Marx S., Dhalapathy S., Anupa S. (2010). Anthropometric analysis of the infraorbital foramen in a south indian population. *Singapore Medical Journal*.

[15] Cisneiros de Oliveira L. C., Silveira M. P., de Almeida Júnior E., Reis F. P., Aragão J. A. (2016). Morphometric study on the infraorbital foramen in relation to sex and side of the cranium in northeastern Brazil. *Anatomy & Cell Biology*.

[16] Macedo V. C., Cabrini R. R., Faig-Leite H. (2009). Infraorbital foramen location in dry human skulls. *Brazilian Journal of Morphological Sciences*.

[17] Ercikti N., Apaydin N., Kirici Y. (2016). Location of the infraorbital foramen with reference to soft tissue landmarks. *Surgical and Radiologic Anatomy*.

[18] Takahashi Y., Kakizaki H., Nakano T. (2011). Infraorbital foramen: horizontal location in relation to ala nasi. *Ophthalmic Plastic and Reconstructive Surgery*.

[19] Song W.-C., Kim S.-H., Paik D.-J. (2007). Location of the infraorbital and mental foramen with reference to the soft-tissue landmarks. *Plastic and Reconstructive Surgery*.

[20] Ekambaram G., Shaik R., Salmani D., Ekambaram G. (2014). A genderwise study on the morphometry of infraorbital foramen and its laterally in dry adult skulls of South Indian population. *International Journal of Medical Science and Public Health*.

[21] Singh R. (2011). Morphometric analysis of infraorbital foramen in Indian dry skulls. *Anatomy and Cell Biology*.

[22] Gupta T. (2008). Localization of important facial foramina encountered in maxillo-facial surgery. *Clinical Anatomy*.

[23] Elsheikh E., Nasr W. F., Ibrahim A. A. S. (2013). Anatomical variations of infraorbital foramen in dry human adult egyptian skulls, anthropometric measurements and surgical relevance. *Otorhinolaryngology Clinics*.

[24] Singh A., Agarwal P., Singh N., Debberma S. (2015). Accessory infraorbital foramen and Morphometric localization of infraorbital foramen. *National Journal of Integrated Research and Medicine*.

[25] Bigoni L., Velemínská J., Brůžek J. (2010). Three-dimensional geometric morphometric analysis of cranio-facial sexual dimorphism in a Central European sample of known sex. *HOMO- Journal of Comparative Human Biology*.

[26] Rossi M., Ribeiro E., Smith R. (2003). Craniofacial asymmetry in development: an anatomical study. *Angle Orthodontist*.

[27] Bhart A., Purani M. G. (2013). Morphometric study of infraorbital foramen in dry human skulls. *National Journal of Integrated Research in Medicine*.

[28] Leo J. T., Cassell M. D., Bergman R. A. (1995). Variation in human infraorbital nerve, canal and foramen. *Annals of Anatomy*.

[29] Kadanoff D., Mutanoff S. T., Jordanov J. (1970). Über die Hauptöffnungen resp. incisurae des Gesichtsschädels. *Morphologisches Jahrbuch*.

[30] Berry A. C. (1975). Factors affecting the incidence of non metrical skeletal variants. *Journal of Anatomy*.

[31] Tezer M., Öztürk A., Akgül M., Gayretli Ö., Kale A. (2011). Anatomic and morphometric features of the accessory infraorbital foramen. *Journal of Morphological Sciences*.

[32] Bressan C., Geuna S., Malerba G. (2004). Descriptive and topographic anatomy of the accessory infraorbital foramen. Clinical implications in maxillary surgery. *Minerva stomatologica*.

[33] Bahrami A., Saman M., Ducic Y. (2016). Duplicate infraorbital nerve—an uncommon anatomical variation. *JSM Oro Facial Surgeries*.

[34] Tubbs R. S., Loukas M., May W. R., Cohen-Gadol A. A. (2010). A variation of the infraorbital nerve: its potential clinical consequence especially in the treatment of trigeminal neuralgia: case report. *Neurosurgery*.

[35] Mohammed A. I., Fatah A. A. (2013). Computed tomographic localization of infraorbital foramen position and correlation with the age and gender of Iraqi subjects. *Journal of Baghdad College of Dentistry*.

[36] Brandao F. H., Machado M. R. C. S., de Aquino J. E. P., Coelho R. G., Pereira S. H. P., Fabi R. P. (2008). The foramen and infraorbital nerve relating to the surgery for external access to the maxillary sinus (CALDWELL-LUC). *International Archives of Otorhinolaryngology, Sao Paulo*.

